# Integrating network pharmacology and experimental validation to investigate the mechanism of Gualou Xiebai Banxia decoction against myocardial ischemia

**DOI:** 10.3389/fcvm.2025.1512791

**Published:** 2025-04-22

**Authors:** Wanjun Zhu, Ping Shao, Ying Ren, Wenxuan Liu, Xiaorui Han, Kexin Zeng, Chunmei Dai, Feifei Liu

**Affiliations:** ^1^ School of Basic Medicine, Jinzhou Medical University, Jinzhou, Liaoning, China; ^2^ Benxi National Engineering Research Center for the Pharmaceutics of Traditional Chinese Medicines Co., Ltd., Benxi, Liaoning, China; ^3^ Institute of Materia Medica, Jinzhou Medical University, Jinzhou, Liaoning, China; ^4^ The First Affiliated Hospital of Jinzhou Medical University, Jinzhou, Liaoning, China

**Keywords:** Gualou Xiebai Banxia decoction, mitochondrial dysfunction, myocardial ischemia, network analysis, PI3K/AKT/NRF2 signaling pathway

## Abstract

**Background:**

Gualou Xiebai Banxia Decoction (GXBD), a traditional Chinese medicine, is used to treat myocardial ischemia (MI). However, the molecular mechanisms underlying its effects remain unclear. This study integrated network pharmacology and experimental validation to investigate the efficacy and potential mechanisms of GXBD in MI treatment.

**Methods:**

Network pharmacology was used to predict the mechanism of action of GXBD in MI. The predicted results were verified using ECG, echocardiography, HE staining, TTC staining, DHE, JC-1, immunofluorescence, and Western blot analysis in an isoproterenol (ISO)-induced MI rat model.

**Results:**

Network pharmacology identified 33 active components in GXBD and 139 potential targets against MI, with the PI3K/AKT signaling pathway playing a key role. Compared to the model group, GXBD improved the activities of BNP, CK-MB, and LDH, ameliorated the general condition and cardiac function, and repaired heart damage in MI rats. GXBD decreased MDA and ROS levels, increased SOD and GSH-Px levels, and protected cardiac tissues from oxidative stress. Moreover, GXBD increased ATP content, mitochondrial membrane potential, and the levels of p-PI3K, p-AKT, nuclear NRF2, and MFN2, while decreasing the levels of cytoplasmic NRF2 and DRP1.

**Conclusion:**

This study suggested that GXBD alleviates myocardial ischemia by ameliorating mitochondrial dysfunction through the PI3K/AKT/NRF2 signaling pathway.

## Introduction

Myocardial ischemia (MI) is a pathological condition characterized by reduced blood perfusion and oxygen supply to the heart, leading to abnormal myocardial energy metabolism and impaired cardiac function (1). With the improvement of living standards, MI has become a common cardiovascular disease (2). Studies have shown that the pathogenesis of MI involves various biological processes, including oxidative stress, mitochondrial dysfunction, apoptosis, and inflammation (3–5). Mitochondrial dysfunction caused by MI significantly contributes to myocardial damage (6). It has been found that after 20–30 min of myocardial ischemia, mitochondria become translucent, with increased membrane gaps, enlarged cristae, and disrupted matrix (7). The increased mitochondrial membrane spacing blocks the efficient transfer and synthesis of ATP, resulting in reduced cardiomyocyte activity and cardiac function (8–10). Currently, nitrates and beta-blockers are commonly used to treat cardiovascular diseases. However, they are prone to adverse reactions such as gastrointestinal issues, abnormal lipid metabolism, and allergies (11). Therefore, finding safer and more effective drugs for MI treatment is a primary task.

Traditional Chinese medicine (TCM) has a long history in China and has shown great advantages in the treatment of cardiovascular diseases, making it an alternative therapeutic strategy for the prevention and treatment of MI (12, 13). Gualou Xiebai Banxia Decoction (GXBD) is a classic prescription recorded in “Jin Kui Yao Lue” by Zhang Zhongjing, a famous Chinese physician from the Han Dynasty (14, 15). It mainly contains flavonoids, triterpenoids, alkaloids, amino acids, and other chemical components (16). For centuries, GXBD has been widely used to treat many cardiovascular disorders, including myocardial infarction, heart failure, and arrhythmias (17). However, the understanding of its molecular mechanisms and signaling targets in cardiovascular diseases is still in the early stages. Therefore, it is important to study the mechanisms of action of GXBD in cardiovascular diseases.

With the rise of network pharmacology, its integral and systematic characteristics have become consistent with the holistic view of TCM and the principles of syndrome differentiation and treatment, leading to its widespread use in research. Based on the interactions among drugs, components, targets, and diseases, combined with biological and drug action networks, this method analyzes and predicts the potential active components and mechanisms of drugs (1, 14). In this study, network pharmacology was used to explore the possible mechanisms of GXBD, and its effects on MI were verified through animal experiments.

## Methods and materials

### Network pharmacology

The three main traditional Chinese medicines comprising GXBD were screened for oral bioavailability (OB) ≥ 30% and drug similarity (DL) ≥ 0.18 using the TCMSP database (https://tcmsp-e.com/). The identified compounds were standardized using the UniProt database (https://www.uniprot.org/). Related targets were searched in the GeneCards database (https://www.genecards.org/), and genes associated with “myocardial ischemia” were identified. Targets with relevance scores higher than the median were selected to improve reliability. The results were collated and merged to remove duplicates and obtain the final targets. A network of active ingredients and key targets was constructed using Cytoscape 3.7.2 software to elucidate the interactions between the active components of GXBD and anti-MI targets. Potential overlapping targets of GXBD and MI-related genes were imported into the STRING platform to construct a protein-protein interaction (PPI) network. The intersection targets were imported into the DAVID database (https://david.ncifcrf.gov/) for Gene Ontology (GO) and Kyoto Encyclopedia of Genes and Genomes (KEGG) enrichment analyses. The results were screened based on the false discovery rate (FDR) and *P* value, and the online platform Weshengxin (http://www.bioinformatics.com.cn/) was used to visualize the results. By analyzing the topological parameters of the network graphs, nodes with degree value, betweenness centrality, and closeness centrality greater than the mean were identified as the core components and targets of GXBD in the treatment of MI.

### Chemicals and reagents

*Trichosanthes*, *Allium macrostemon*, and *Pinellia* ternata were purchased from Anguo Juyaotang Pharmaceutical Co., Ltd. (batch numbers: 2,308,004, 2,309,004, and 2,311,002, respectively). White wine was purchased from Guyue Longshan Shaoxing Wine Co. Ltd. Three herbs, Gualou (*Trichosanthes*), Xiebai (*Allium macrostemon*), and Banxia (*Pinellia ternata*), were taken in the following amounts: 60.6 g of Gualou, 41.4 g of Xiebai, and 34.5 g of Banxia. The herbs were soaked in 2000 ml of white wine for 30 min. The mixture was heated in a heating jacket, boiled to 800 ml, and maintained at a slight boil for 150 min. The mixture was filtered twice through a 300-mesh cloth while still hot. The solution was concentrated and cooled to room temperature. Afterwards, 20 g of the test article was weighed using precision, placed in a dry and constant weight evaporating dish, and evaporated to dryness. The sample was dried at 105°C for 3 h in an electric blast drying oven, removed, cooled, weighed, and the solid content was calculated to be 39.77%. Propranolol was purchased from Jiangsu Yabang Apson Pharmaceutical Co., Ltd. (batch number: H32020133, Jiangsu, China) and isoprenaline was purchased from Sigma Corporation (United States). TTC staining solution was obtained from Beijing Solepol Co. (Beijing, China). Lactate dehydrogenase (LDH, A020-2-1), adenosine triphosphate (ATP, A095-1-1), superoxide dismutase (SOD, A001-3-2), glutathione peroxidase (GSH-px, A005-1-2), and malondialdehyde (MDA, A003-1-2) assay kits were obtained from Nanjing Jianjian Biological Co. (Nanjing, China). Brain natriuretic peptide (BNP, H166-1-1) and creatine kinase isoenzyme (CK-MB, H197-1-1) were purchased from R&D Systems. RIPA lysate, phosphatase inhibitor, PMSF solution, DHE, Nuclear and Cytoplasmic Protein Extraction kits, and JC-1 kits were obtained from Beyotime Biotechnology (Shanghai, China). The BCA protein quantification kit was purchased from Beijing Dingguo Changsheng Biotechnology Co. (Beijing, China). PI3K (A0982, 1:1,000 dilution), p-PI3K (AP0427, 1:1,000 dilution), AKT (A17909, 1:1,000 dilution), p-AKT (AP1068, 1:1,000 dilution), NRF2 (A11159, 1:1,000 dilution), HO-1 (A23650, 1:1,000 dilution), DRP1 (A2586, 1:1,000 dilution), MFN2 (A12771, 1:400 dilution), and β-actin (AC026, 1:100,000 dilution) antibodies, as well as horseradish peroxidase (HRP)-labeled goat anti-rabbit IgG antibody (AS014, 1:5,000 dilution), were purchased from ABclonal Technology Co., Ltd. (Wuhan, China). Enhanced chemiluminescence kits were obtained from Biosharp, Inc. (Hefei, China).

### Animals experiments

Male Sprague–Dawley rats, weighing 180–220 g, were purchased from the Animal Experiment Center of Jinzhou Medical University. All rats were housed in a 12 h light/dark cycle at a temperature of 23 ± 1°C with free access to food and water. Animal studies were conducted in accordance with the standards and guidelines established by the Guide for the Care and Use of Laboratory Animals formulated by the National Institutes of Health (China).

Rats were randomly divided into six experimental groups (*n* = 10 per group): control, model, propranolol (3 mg/kg/d), low-dose, medium-dose, and high-dose GXBD (2.4 g/kg/d, 4.8 g/kg/d, 9.6 g/kg/d, respectively). The dosages of propranolol and GXBD were calculated based on the daily doses commonly used in humans, converted for rats according to the third edition of “Methodology of Pharmacological Research on Traditional Chinese Medicine” edited by Chen Qi (18). The clinical dosage of GXBD was selected as the medium dose, with the low dose being half of the medium dose and the high dose being twice the medium dose. The GXBD groups were administered GXBD, the model and control groups were administered normal saline, and the propranolol group was administered propranolol. The corresponding treatments were administered by gavage once daily for 14 days. All groups except the control group were injected subcutaneously in multiple spots on the back with ISO 20 mg/kg/d 1 h after the last administration on the 13th day for 2 days to establish the ISO-induced MI injury model (19). The control group was injected subcutaneously with the same dose of normal saline. All rats were anesthetized by intraperitoneal injection of 400 mg/kg urethane, and blood was collected from the abdominal aorta using a blood collection needle and negative pressure tube. Blood samples were centrifuged at 3,500 rpm for 15 min at 4°C. The supernatant was collected and stored at −80°C. Rats were euthanized under anesthesia, their hearts were quickly removed, and heart weights were recorded (the cardiac weight index was obtained by dividing heart weight by body weight). One part of the heart was fixed in 4% paraformaldehyde, one part was washed with pre-cooled PBS to remove all blood for TTC staining, and the other part was immediately stored at −80°C until further analysis. All animal experiments were approved by the Animal Ethics Committee of Jinzhou Medical University (No. 240096).

### Electrocardiogram (ECG)

After the last injection of ISO, the rats were fasted for 12 h, weighed, and anesthetized by intraperitoneal injection of urethane. After 5 min of anesthesia induction, the rats were placed in the supine position. Needle electrodes were inserted subcutaneously from the right forelimb to the left hind limb and connected to the BL-420N biological function experimental system to record the standard lead II electrocardiogram.

### Echocardiography

After anesthesia, the rats were positioned supine, and their chests were fully exposed. Excess fur on the rats' chests was removed using dehairing cream. The MyLab 30CV ultrasound system (Biosound Esaote, Indianapolis, IN, USA) with a 15-MHz transducer probe was used to perform M-mode echocardiographic measurements. Parasternal long axis and short axis images were obtained in short and long axes in M-mode for quantification. Parameters included left ventricular ejection fraction (EF), fractional shortening (FS), left ventricular posterior wall thickness at end-diastole (LVPED), and left ventricular posterior wall thickness at end-systole (LVPWS).

### Histopathological and TTC tests

Paraffin-embedded sections of cardiac tissue samples were stained with hematoxylin and eosin (H&E). Briefly, rat left ventricular tissues were fixed with 4% paraformaldehyde, treated with a gradient of ethanol and xylene, and embedded in paraffin. The embedded tissue was cut into 4–5 μm sections, treated with xylene and gradient ethanol, and stained with haematoxylin‒eosin (H&E) staining solution. Finally, photographs were taken under a light microscope.

Fresh heart tissue from rats was stained with TTC. The samples were washed, transferred to a pre-cooled PBS solution, and frozen at −20°C for 30 min. Subsequently, 2 mm sections were cut and immersed in a 2% TTC solution at 37°C in the dark for 30 min. The container was shaken every 5 min to ensure uniform staining. The infarcted areas appeared white, whereas the uninfarcted areas appeared red. Infarct area (infarct area/total left ventricular area ×100%) was calculated using ImageJ software.

### Biochemical index determination

Serum lactate dehydrogenase (LDH), adenosine triphosphate (ATP), superoxide dismutase (SOD), glutathione peroxidase (GSH-px), and malondialdehyde (MDA) levels were detected using commercial kits. The supernatant was used to evaluate brain natriuretic peptide (BNP) and creatine kinase isoenzyme (CK-MB) levels according to the ELISA kit instructions.

### Reactive oxygen species (ROS) detection

Heart specimens were cut into 5 μm-thick sections, washed with xylene, dehydrated, stained with DHE (10 μM) staining solution, incubated at 37°C in the dark for 30 min, and washed twice with PBS. Images were taken using a fluorescence microscope.

### Measurement of mitochondrial membrane potential

Paraffin-embedded sections of the hearts were washed with xylene and dehydrated. The sections were stained with JC-1 (5 mmol/L) at 37°C in the dark for 30 min and then washed with PBS. After washing, the slides were sealed with an anti-fluorescence burst sealer and photographed under a fluorescence microscope.

### Immunofluorescence staining

The paraffin sections of the hearts were washed with xylene, dehydrated, and permeabilized for 10 min with Triton X-100 (0.3%). The sections were incubated with 10% goat serum for 1 h at room temperature, followed by incubation with the primary antibody NRF2 (1:100) overnight at 4°C. Subsequently, the tissue sections were washed thrice with PBS and incubated for 2 h with a fluorescent secondary antibody. Finally, DAPI sealant was added for 5 min. Images were acquired via fluorescence microscopy.

### Western blot analysis

Heart tissue (100 mg) was homogenized in 1 ml RIPA buffer containing 1% PMSF and 2% phosphatase inhibitor on ice, and then centrifuged at 12,000 rpm for 10 min at 4°C. The nuclei and cytoplasm were extracted according to the corresponding steps. The supernatant was obtained and the protein concentration was determined using a BCA protein kit. Proteins were separated by sodium dodecyl sulfate-polyacrylamide gel electrophoresis and transferred onto polyvinylidene fluoride (PVDF) membranes. PVDF membranes were blocked with 5% skim milk powder at room temperature and subsequently incubated with primary antibodies against PI3K, p-PI3K, AKT, p-AKT, NRF2, HO-1, DRP1, and MFN2 overnight at 4°C. After washing three times, the membranes were incubated with an HRP-conjugated antibody for 1 h. PVDF membranes were imaged using an enhanced chemiluminescence (ECL) system. Gray-scale analysis was performed using ImageJ software.

### Statistical analysis

All results were expressed as the mean ± standard deviation (SD) and were analyzed via SPSS 26.0. At least three replications of each experiment were conducted. To evaluate statistical significance, experiments that conformed to normal distribution data were analyzed by one-way ANOVA. Otherwise, Student's *t*-test was used. Pairwise comparisons that satisfied the homogeneity of variance were performed using the LSD method, while those that did not satisfy the homogeneity of variance were performed using the Games-Howell method. *P* < 0.05 was considered statistically significant.

## Results

### Network pharmacology forecasted the potential mechanism of GXBD on MI

**Figure 1:**
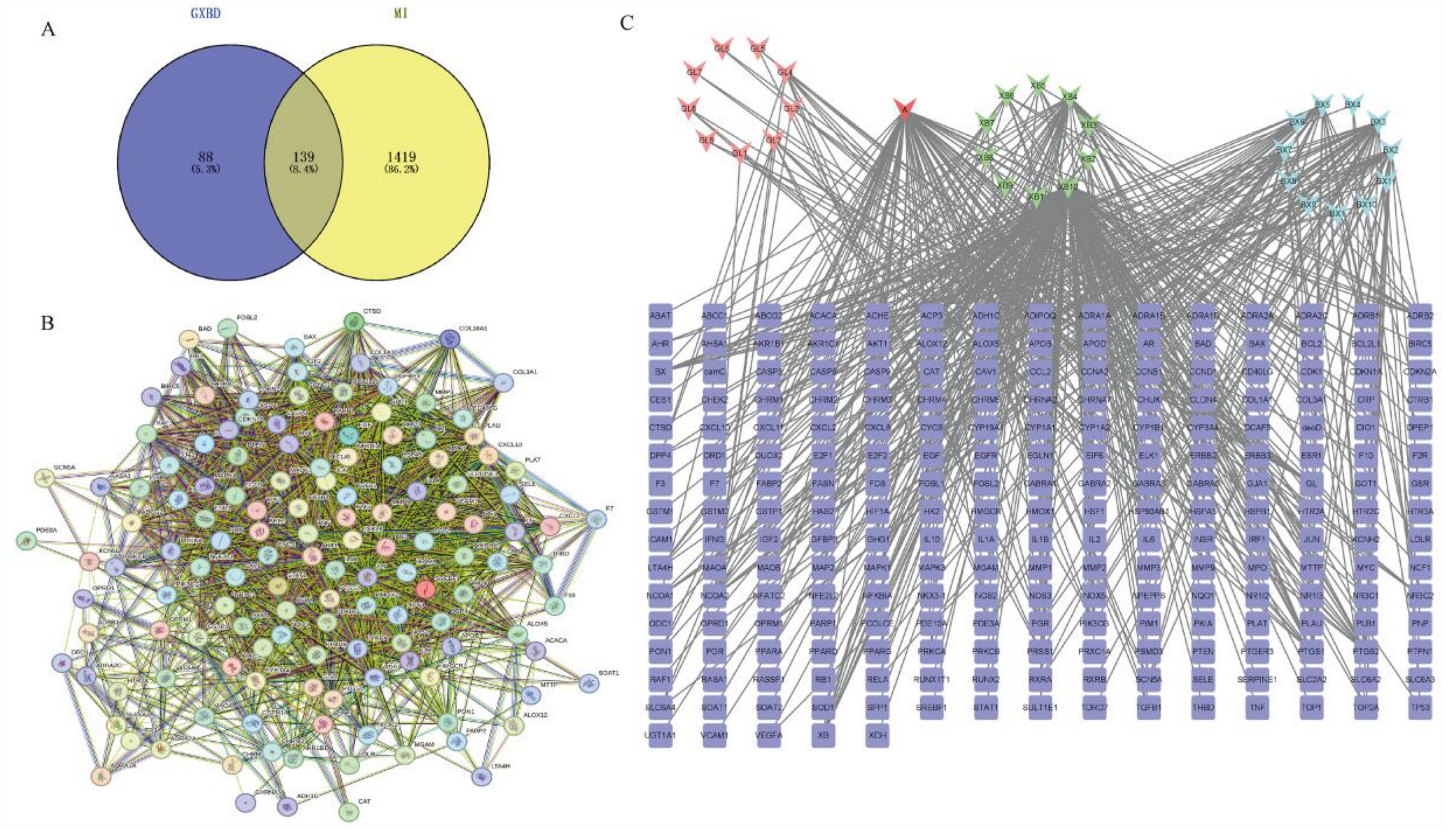
Overlapped targets screen. **(A)** Overlapping targets of overlapping GXBD and MI. Blue circle represented GXBD targets and yellow circle represented MI targets. The overlapping region was the target of GXBD against MI. **(B)** The protein-protein interaction (PPI) network of targets. PPI network of potential targets of GXBD against MI. **(C)** Relationship network between potential therapeutic targets against MI and active components of GXBD (The light red triangle represented the active components of Gualou, the green triangle represented the active components of Xiebai, the blue triangle represented the active components of Banxia, the dark red triangle represented the common components of Banxia and Xiebai, and the blue square represented the target of Gualou Xiebai Banxia Decoction for anti-myocardial ischemia).

**Figure 2:**
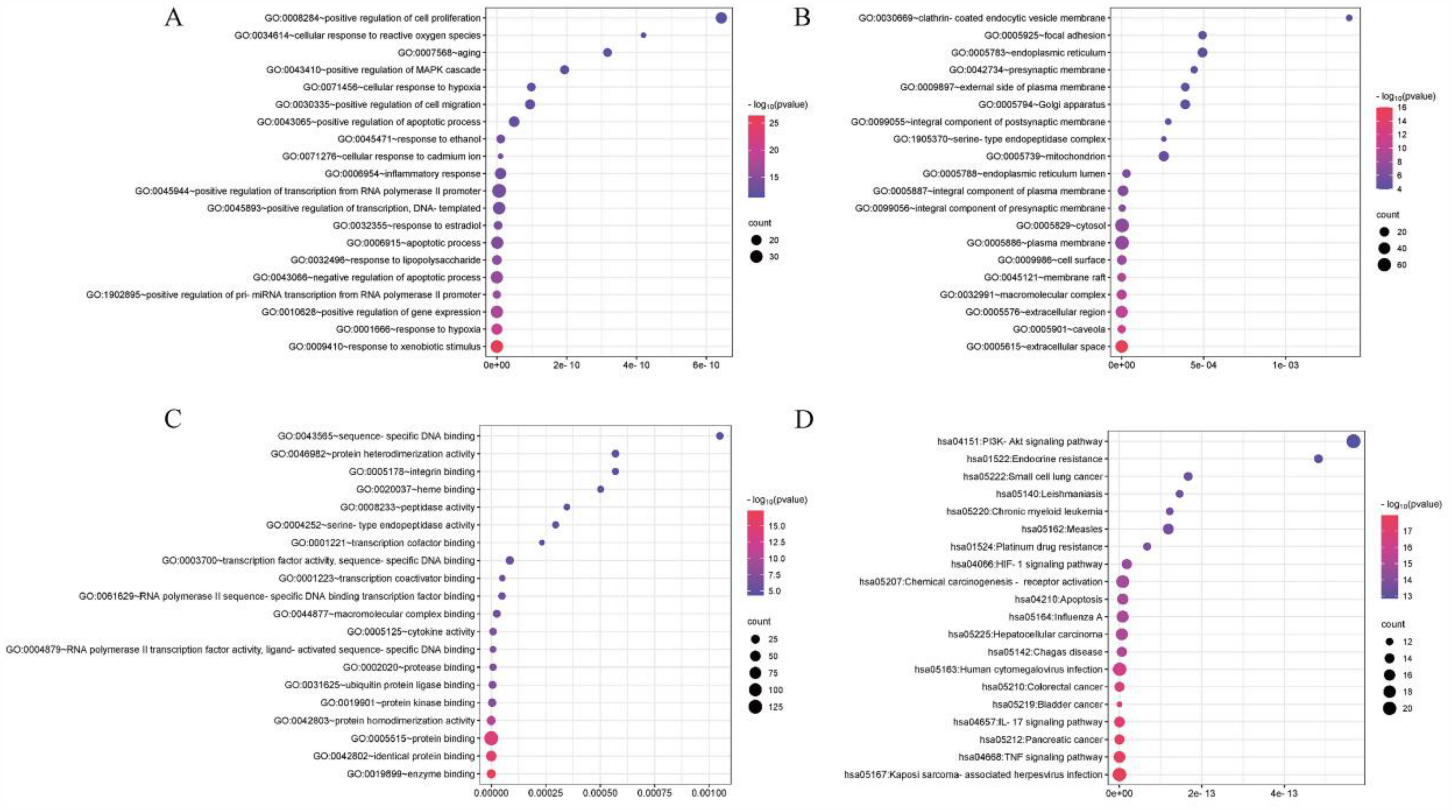
Results of GO enrichment and KEGG pathway analyses of GXBD targets. **(A)** Biological process. **(B)** Cellular component. **(C)** Molecular function. **(D)** KEGG pathway. (The GO terms are located on the *Y*-axis, and the numbers on the *X*-axis indicate the degree of enrichment. Red indicated high reliability, while blue indicated low reliability.).

Thirty-three different types of GXBD active components were collected from the TCMSP database, including 9 types of Gualou, 11 types of Xiebai, and 13 types of Banxia. A total of 227 targets were collected from the TCMSP. The gene targets were analyzed, duplicates were removed, and 1,558 disease targets associated with MI were obtained. The predicted set of 227 potential targets and 1,558 MI disease genes were intersected to determine the set of potential targets for GXBD in MI treatment, yielding 139 targets for GXBD in MI treatment (Figure 1A). The intersecting targets of GXBD were inserted into the STRING platform to construct a PPI network of potential targets of GXBD for MI (Figure 1B). A network of relationships between the potential therapeutic targets against MI and the active ingredients of GXBD was established (Figure 1C). Finally, enrichment analysis of molecular functions, biological processes, cellular components, and KEGG pathways was performed for the 139 possible targets. GO analysis results showed that the biological processes of these targets mainly involved the positive regulation of cell proliferation, cellular response to reactive oxygen species, and aging (Figure 2A). The cellular components were mainly related to the clathrin-coated endocytic vesicle membrane, focal adhesion, and the endoplasmic reticulum (Figure 2B). The main molecular functions were sequence-specific DNA binding, protein heterodimerization, and integrin binding (Figure 2C). The results of KEGG functional enrichment analysis showed that the active components of GXBD affected MI mainly through pathways in pancreatic cancer, the PI3K-AKT signaling pathway, and the IL-17 signaling pathway (Figure 2D).

### GXBD ameliorated cardiac function in MI rats

**Figure 3:**
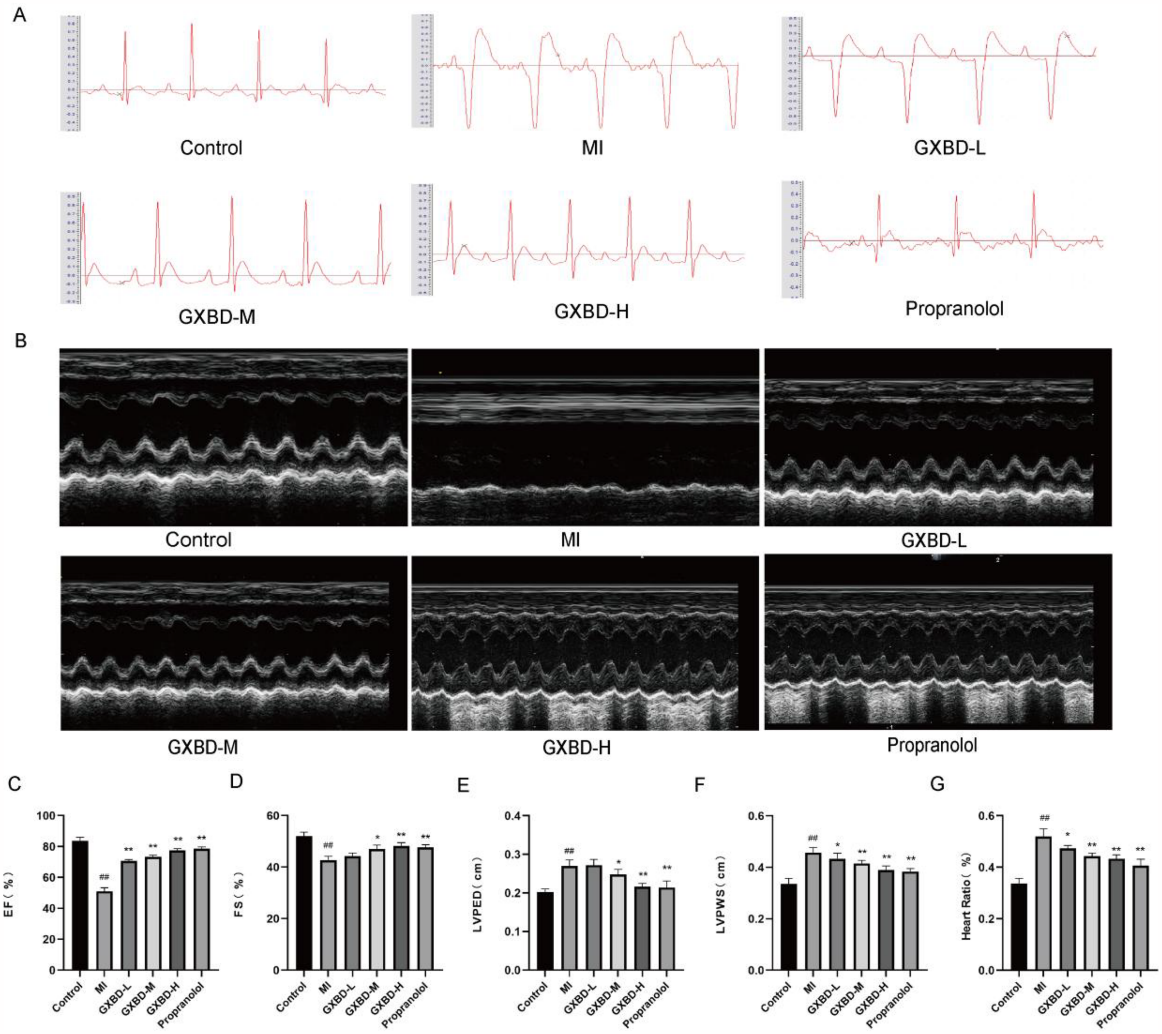
GXBD ameliorated cardiac function in MI rats. **(A)** Electrocardiogram of rats. **(B)** Representative M-mode echocardiography images. **(C–F)** EF ejection fraction, FS fractional shortening of the left ventricular diameter, LVPED diastolic left ventricular internal diameter, LVPWS systolic left ventricular posterior wall thickness. **(G)** Heart-to-Body-Weight ratio. All data were expressed as mean ± SD, *n* = 5. Compared with the control group, ^#^*P* < 0.05, ^##^*P* < 0.01. Compared with the MI model group, *P < 0.05, **P < 0.01.

After the first subcutaneous injection of isoproterenol in the back, all rats showed decreased activity, increased oral and nasal secretions, shortness of breath, and a slow response, which may be related to the isoproterenol-activated β receptor. There was no significant difference in initial body weight between the treatment groups. Compared with the control group, the S-T segment of the electrocardiogram in the model group was elevated, indicating that the rat model of MI was successfully reproduced. Compared to the model group, the S-T segment of the ECG in each dose of GXBD and propranolol groups decreased (Figure 3A).

Echocardiography results showed that cardiac function was significantly decreased in the model group; however, EF, FS, LVPED, and LVPWS were increased in the three GXBD dose groups and propranolol group, suggesting enhanced cardiac function in these rats (Figures 3B–F).

Additionally, the cardiac index was significantly higher in the MI model group compared to the control group. The cardiac index was reduced to varying degrees in each treatment group compared to the model group (Figure 3G).

### GXBD improved cardiac injury in MI rats

**Figure 4:**
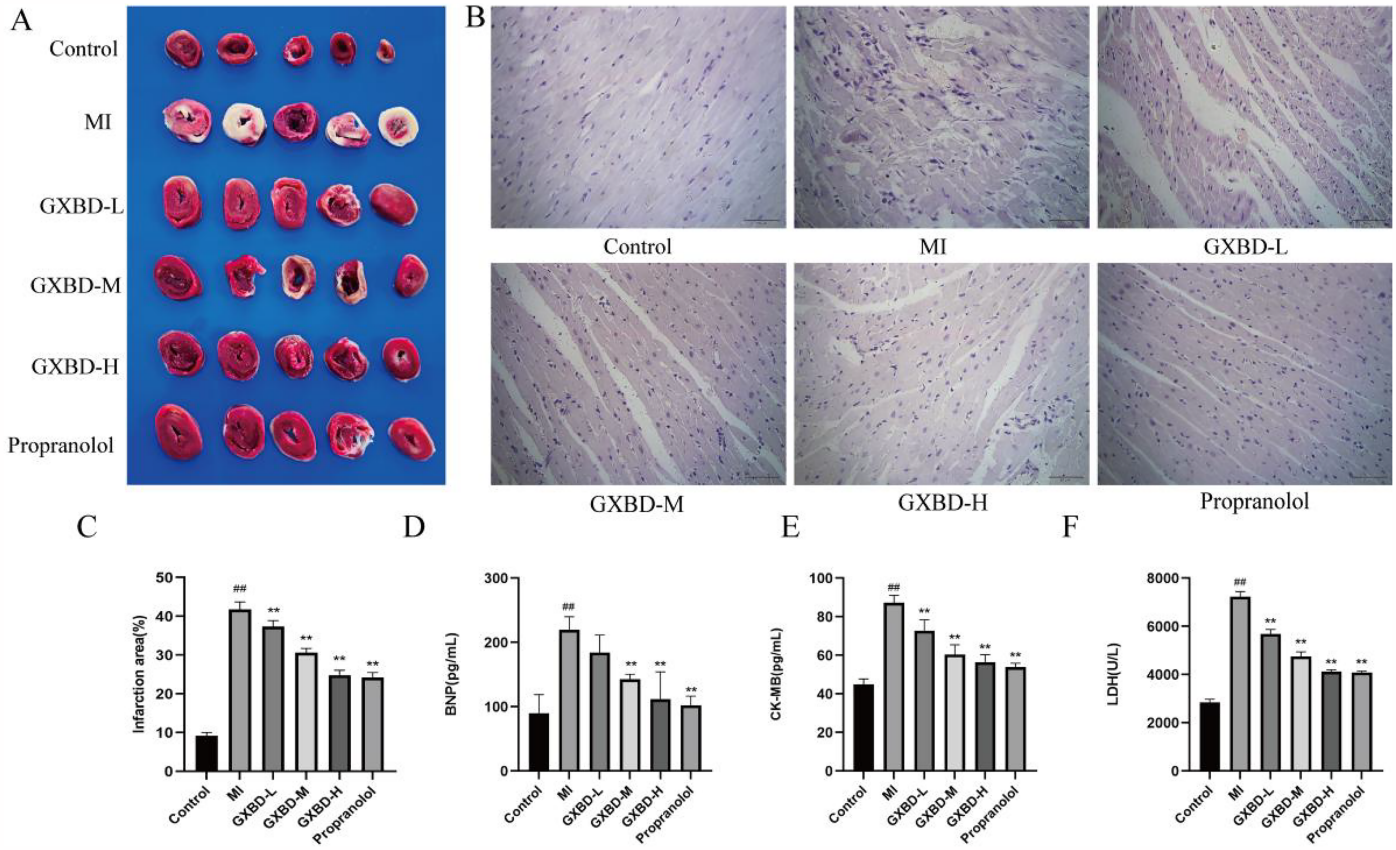
GXBD improved cardiac injury in MI rats. **(A)** Cardiac ischemia was detected by TTC staining. **(B)** H&E staining was used to observe the pathological changes of rat heart tissue (×400). **(C)** Infarction area. **(D)** brain natriuretic peptide. **(E)** creatine kinase isoenzyme. **(F)** lactate dehydrogenase. All data were expressed as mean ± SD, *n* = 5. Compared with the control group, ^#^*P* < 0.05, ^##^*P* < 0.01. Compared with the MI model group, *P < 0.05, **P < 0.01.

Myocardial injury was evaluated by TTC staining. No myocardial infarction was observed in the control group. The model group had obvious infarct areas compared to the control group. Infarct size was reduced in each treatment group compared to the model group (Figure 4A).

Hematoxylin-eosin (H&E) staining was used to evaluate morphological changes in cardiomyocytes. In the control group, myocardial tissue cross striations were arranged in an orderly, uniform manner with consistent coloration of healthy cardiomyocytes. Histological analysis of HE-stained heart sections from the model group showed inflammatory cell infiltration, myocardial necrosis, and structural disorder, indicating myocardial injury. The degree of myocardial injury was reduced to a certain extent in each treatment group compared to the model group (Figure 4B).

BNP, CK-MB, and LDH were used as marker enzymes for myocardial injury (20). Compared to the control group, BNP, CK-MB, and LDH levels were significantly increased in the model group (*P* < 0.01). Serum levels of these markers were decreased to varying degrees in the propranolol group compared to the model group. BNP levels were decreased in the middle and high dose GXBD groups, while CK-MB and LDH levels were decreased to some extent in all GXBD dose groups (*P* < 0.01) (Figures 4C–F).

### GXBD reduced oxidative stress in MI rats

**Figure 5:**
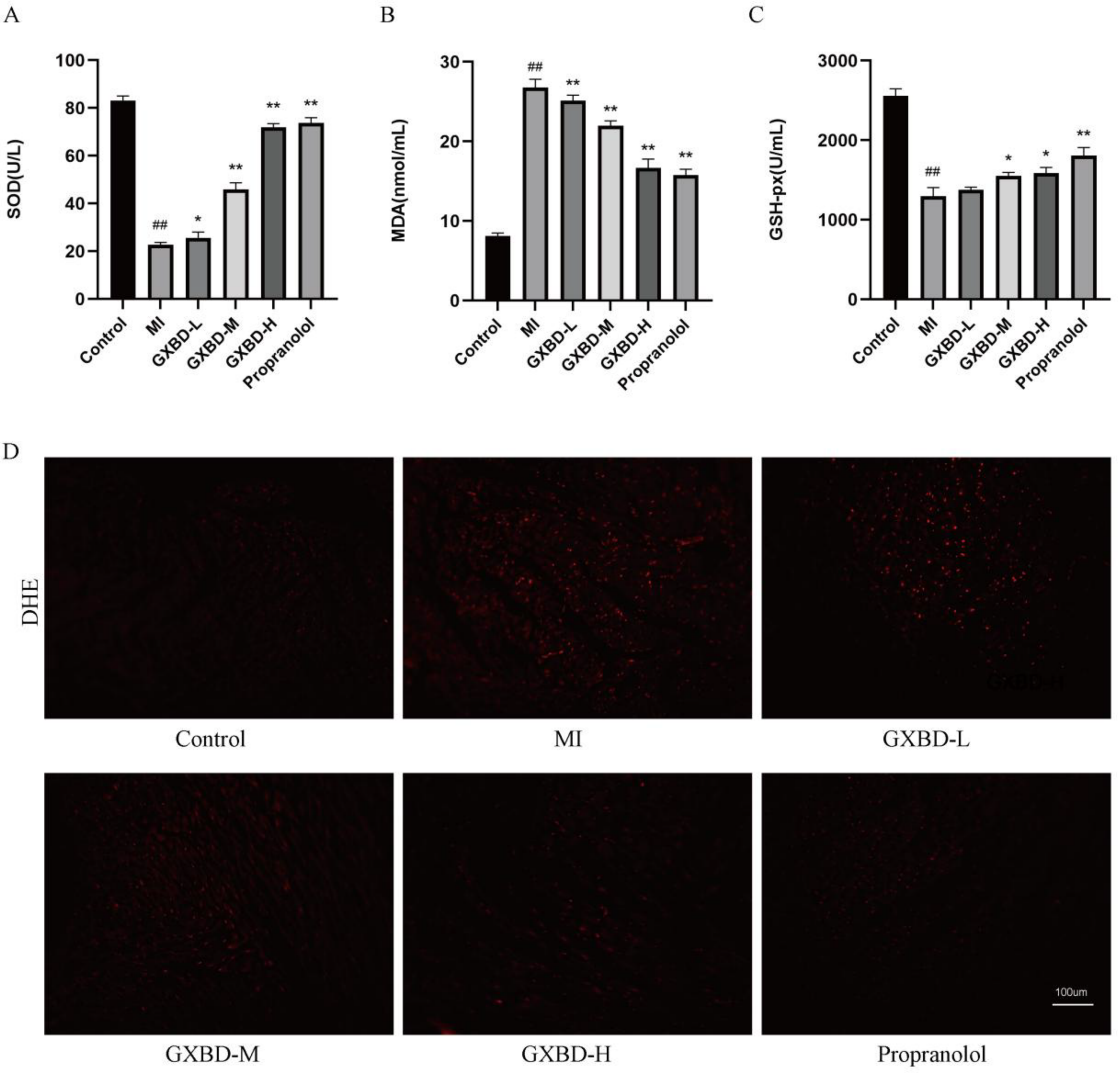
GXBD reduced oxidative stress in MI rats. **(A)** superoxide dismutase. **(B)** malondialdehyde. **(C)** glutathione peroxidase. **(D)** ROS content in rat myocardial tissue (×200). All data were expressed as mean ± SD, *n* = 5. Compared with the control group, ^#^*P* < 0.05, ^##^*P* < 0.01. Compared with the MI model group, *P < 0.05, **P < 0.01.

Oxidative stress is an important mechanism of MI injury. SOD and GSH-px levels were significantly decreased (*P* < 0.01), while MDA content was significantly increased (*P* < 0.01) in the model group compared to the control group. SOD and GSH-px levels were increased and MDA content was decreased to varying degrees in the propranolol group compared to the model group. SOD levels were increased and MDA content was reduced in all GXBD dose groups. Additionally, GSH-px levels were decreased in the middle and high dose GXBD groups (*P* < 0.05 or *P* < 0.01) (Figure 5A–C).

ROS levels were significantly higher in the model group than the control group. ROS levels were significantly reduced in the treatment groups after administration compared to the model group (Figure 5D).

### GXBD improved mitochondrial function in MI rats

**Figure 6:**
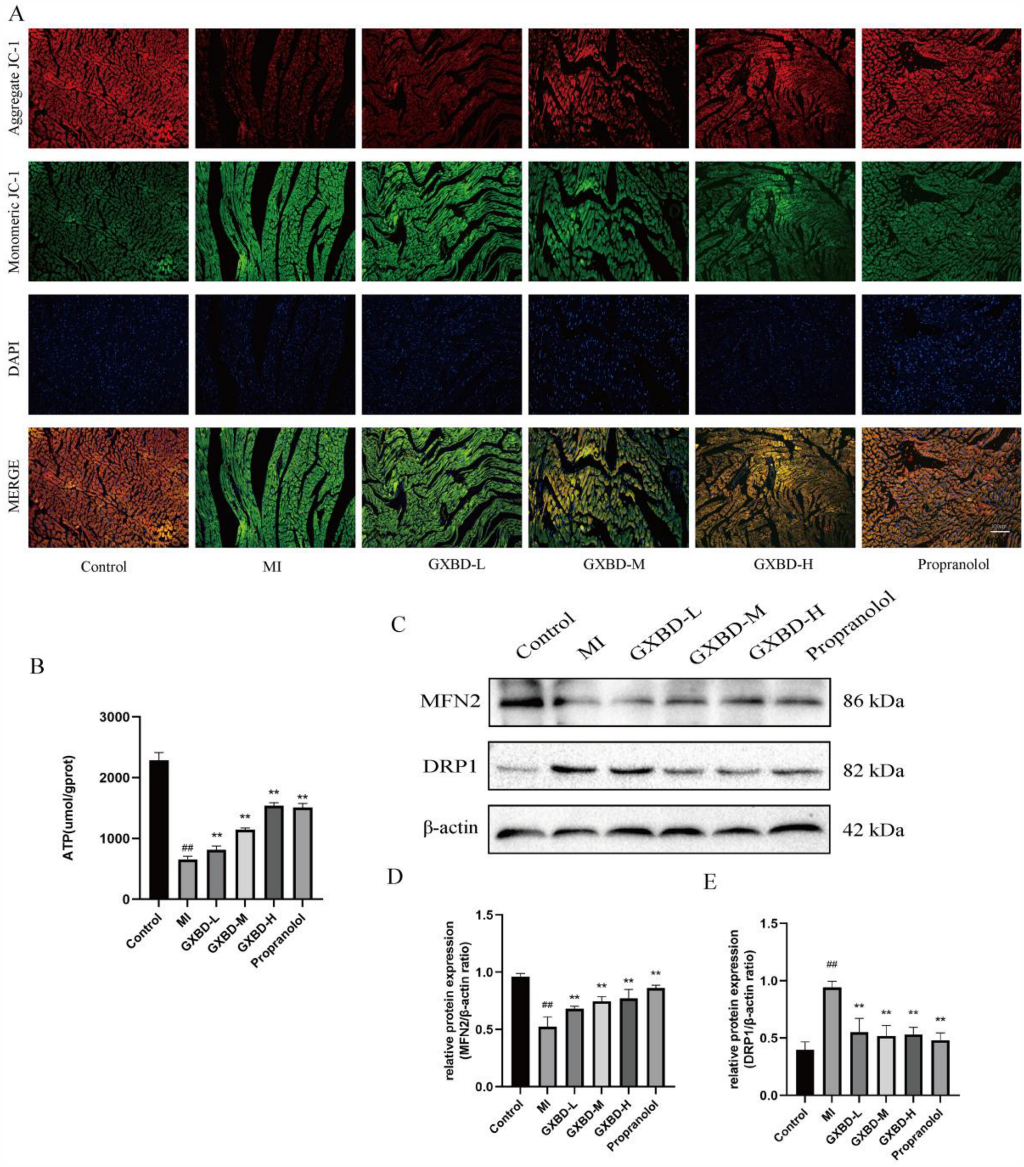
GXBD improved mitochondrial function in MI rats. **(A)** Typical cardiac images drawn in cardiac sections using JC-1 staining (×200). **(B)** ATP. **(C)** MFN2 and DRP1 protein expression in cardiac tissues of rats. **(D)** MFN2/β-actin ratio. **(E)** DRP1/β-actin ratio. All data were expressed as mean ± SD, *n* = 5. Compared with the control group, ^#^*P* < 0.05, ^##^*P* < 0.01. Compared with the MI model group, *P < 0.05, **P < 0.01.

To assess the effects of GXBD on mitochondria, ATP content, mitochondrial membrane potential, and related protein expression were examined. ATP content and mitochondrial membrane potential were significantly reduced in the model group compared to the control group (*P* < 0.01). These parameters were improved to varying degrees in all treatment groups compared to the model group (Figures 6A,B). Additionally, each treatment group increased MFN2 protein expression and reduced DRP1 expression. These results indicate that GXBD improved mitochondrial function in MI rats (Figures 6C–E).

### GXBD improved MI by activating the PI3K/AKT/NRF2 signaling pathway

**Figure 7:**
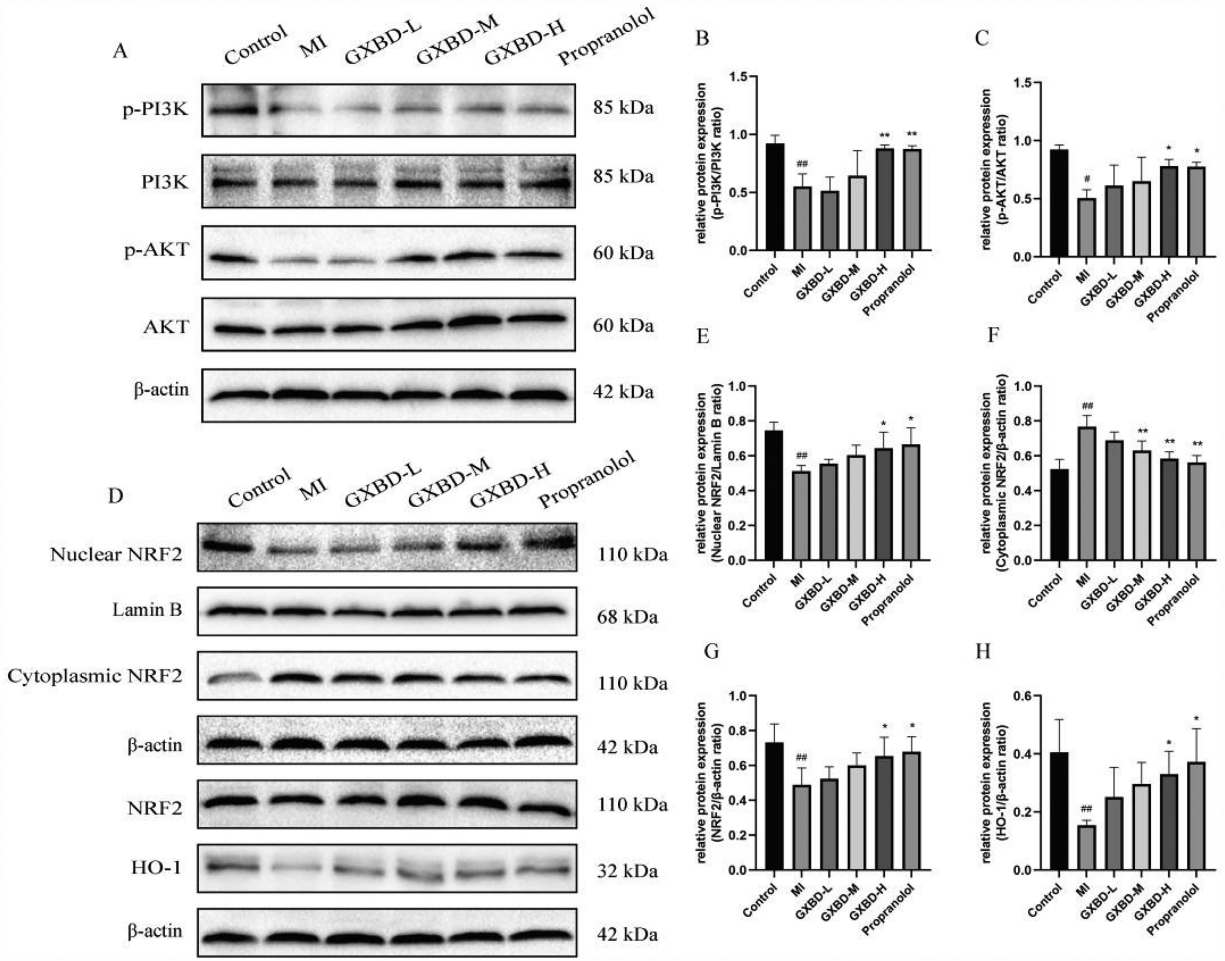
Effect of GXBD on PI3K/AKT/NRF2 pathway protein expression in cardiac tissue of rats with MI. **(A)** p-PI3K, PI3K, p-AKT and AKT protein expression in cardiac tissues of rats. **(B)** p-PI3K/PI3K ratio. **(C)** p-AKT/AKT ratio. **(D)** Nuclear NRF2, Cytoplasmic NRF2, NRF2 and HO-1 protein expression in cardiac tissues of rats. **(E)** NRF2/Lamin B ratio. **(F)** Cytoplasmic NRF2/β-actin ratio. **(G)** NRF2/β-actin ratio. **(H)** HO-1/β-actin ratio. All data were expressed as mean ± SD, *n* = 3. Compared with the control group, ^#^*P* < 0.05,^##^*P* < 0.01. Compared with the MI model group, *P < 0.05, **P < 0.01.

Compared to the control group, p-PI3K, p-AKT, Nuclear NRF2, and HO-1 protein expression decreased, while Cytoplasmic NRF2 expression increased in the ISO-induced model group. The high dose GXBD and propranolol groups showed increased p-PI3K, p-AKT, Nuclear NRF2, NRF2, and HO-1 protein expression compared to the model group. The middle, high dose GXBD, and propranolol groups showed decreased Cytoplasmic NRF2 protein expression (Figure 7).

**Figure 8:**
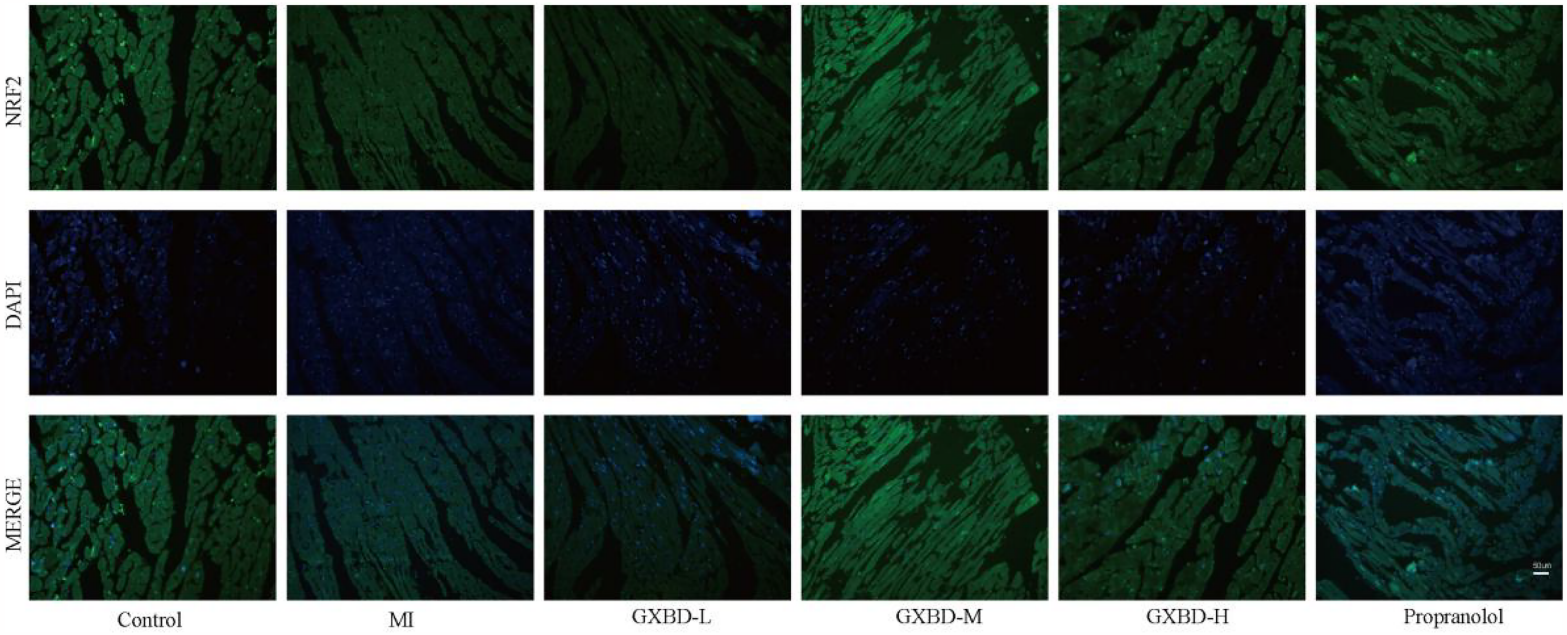
Effects of GXBD on nuclear transcription of NRF2 in MI rats (×400) (*n* = 5).

Additionally, immunofluorescence staining showed that after pretreatment with GXBD and propranolol, NRF2 was mainly located in the nucleus compared to the model group, indicating that GXBD and propranolol may activate NRF2 nuclear translocation (Figure 8).

These results suggest that GXBD and propranolol activate the PI3K/AKT/NRF2 signaling pathway in the heart.

## Discussion

Myocardial infarction is a common acute and critical clinical disease with increasing morbidity and mortality in recent years. GXBD is a classic prescription for treating phlegm, blood stasis, and chest obstruction, and is widely used in modern medicine for the prevention and treatment of cardiovascular diseases. This study predicted the potential mechanism of GXBD treatment using network pharmacology and explored its protective effect and underlying mechanism in animal experiments. The results showed that GXBD could reduce infarct size after MI and improve cardiac function, potentially by activating the PI3K/AKT/NRF2 pathway to improve mitochondrial dysfunction and reduce myocardial injury.

Natural medicines are valuable resources for finding useful drugs to treat various diseases (21, 22). GXBD is a classic formula recorded in the Synopsis of the Golden Chamber by Zhang Zhongjing. The mechanism of modern medicine against cardiovascular diseases still needs further exploration. Network analysis has the characteristics of completeness and systematization, building a bridge for studying the interaction between different drugs. In this study, network pharmacology identified 33 active components in GXBD, 139 potential targets against MI, and highlighted the PI3K/AKT signaling pathway as playing an important role in MI. Related studies have confirmed that components such as baicalein and quercetin play an irreplaceable role in the treatment of myocardial infarction with GXBD. Li et al. found that baicalein may prevent and protect against myocardial ischemia through antioxidant, anti-inflammatory, and anti-apoptotic effects (23). Wang et al. demonstrated that quercetin promoted endogenous energy production and improved mitochondrial dysfunction after myocardial ischemia, thereby reducing myocardial hypoxic injury (24). Furthermore, we chose the PI3K/AKT signaling pathway from KEGG for experimental validation, which is demonstrated to reduce the degree of cardiac damage.

Myocardial ischemia is induced in rats by two subcutaneous injections of isoproterenol (ISO) at a 24 h interval, which has become a reliable model for simulating human myocardial infarction due to its simplicity, non-invasiveness, and lower mortality. Recently, Elshaer A et al. found that the ISO-induced MI model showed the highest level of reliability in simulating human MI by comparing three different methods of inducing MI in rats: surgical approach and subcutaneous injection of ISO on two consecutive days (25). Elevation of the ST segment on the electrocardiogram and reduced cardiac function assessed by echocardiography have been recognized as the most direct ways to identify myocardial ischemia. In this study, pretreatment with GXBD given for 14 consecutive days significantly reduced ST-segment elevation and improved cardiac function in rats with MI, indicating that GXBD has a protective effect on ISO-induced myocardial ischemia.

Brain natriuretic peptide (BNP), creatine kinase-MB (CK-MB), and lactate dehydrogenase (LDH) are identified as essential serum biomarkers for the diagnosis of MI (26). The abnormal myocardial energy metabolism seen during MI elevates the BNP level in the serum (27). Myocardial ischemic injury can lead to increased permeability of cardiomyocyte membranes, leakage of CK-MB from damaged myocardial tissue into the bloodstream, and excessive release of LDH (28). The study showed that administration of GXBD reduced myocardial infarct area, heart/body weight ratio, serum BNP, CK-MB, and LDH activities and attenuated ISO-induced pathologic injury. These results suggest that GXBD has a cardioprotective effect by preventing cardiomyocyte injury, attenuating myocardial necrosis, inflammatory cell infiltration, myocardial fiber rupture, and stabilizing myocardial cell membranes, thereby decreasing serum BNP, CK-MB, and LDH activities.

ISO is capable of inducing excessive Ca^2+^ inward flow, resulting in enhanced myocardial contraction, which in turn increases oxygen demand and leads to electron leakage in the respiratory chain (19). The released free electrons react with molecular oxygen to form reactive oxygen species (ROS). ROS can oxidize mitochondrial proteins, leads to imbalance in mitochondrial dynamics (29). Meng et al. showed that ROS scavengers inhibit dynamin-related protein 1 (Drp1)-mediated mitochondrial fission and promote mitofusin 2 (MFN2)-mediated mitochondrial fusion, ultimately protecting cardiomyocytes (30). Excessive ROS can also induce mitochondrial permeability transition pore (MPTP) opening, full depolarization of the mitochondrial inner membrane, collapse of the inner membrane potential, and reduction of ATP synthesis, leading to mitochondrial dysfunction (31, 32). ATP synthesis is reduced, and the cells undergo anaerobic glycolysis to produce excess pyruvate, leading to cardiomyocyte injury. Insufficient oxygen supply in the blood after cardiomyocyte injury leads to myocardial infarction. Inhibition of the expression of mitochondrial fission-related proteins, such as DRP1, mitochondrial fission factor (Mff), and MFN2, plays a cardioprotective role during MI by reducing infarct size and improving left ventricular dysfunction (33–37). Our study showed that GXBD significantly reduced the level of ROS, increased ATP content and mitochondrial membrane potential, and reversed the changes in MFN2 and DRP1 in the hearts of rats with MI, suggesting that GXBD exerts a protective effect by maintaining mitochondrial function and improving MI in rats.

Transcription factor NF-E2-related factor 2 (NRF2) is bound by its cytosolic negative regulator, Kelch-like ECH-associated protein 1 (Keap1), which hinders its entry into the nucleus. In the ISO injection stimulation state, mitochondrial dysfunction produces excess ROS and lipid peroxide malondialdehyde (MDA). NRF2 evades Keap1, translocates to the nucleus, and binds to the antioxidant response element (ARE) to activate various genes such as heme oxygenase-1 (HO-1), superoxide dismutase (SOD), and glutathione peroxidase (GSH-px) involved in antioxidant defenses to protect cells from oxidative stress (38). NRF2 knockout mice fed a high-fat diet were more prone to elevated ROS levels and endothelial dysfunction than wild-type mice (39). The present study results showed that GXBD treatment promoted NRF2 nuclear translocation, increased HO-1 expression, increased levels of SOD and GSH-px, and decreased levels of MDA, indicating that GXBD can reduce oxidative stress in MI through the NRF2/HO-1 axis.

Phosphatidylinositol 3-kinase (PI3K)/protein kinase B (AKT) acts as an upstream regulator of NRF2-mediated antioxidant responses. PI3K/AKT and its specific inhibitors can regulate cellular defense by inhibiting AKT phosphorylation and subsequent activation of NRF2/HO-1 expression (40, 41). The results showed that GXBD treatment upregulated phosphorylation of PI3K and AKT. Early pretreatment of human renal tubular epithelial cells showed significant protection against hypoxia/reoxygenation (H/R) injury through activation of the PI3K/AKT pathway. GXBD may ameliorate ISO-induced myocardial ischemia by modulating the PI3K/AKT/NRF2 signaling pathway. However, further validation in clinical scenarios is necessary. Limitations include the limited generalizability of ISO-induced models to human myocardial infarction and the fact that the optimal dose and duration of treatment in humans are unknown. In subsequent trials, we will provide additional scientific support for the clinical application of GXBD by studying downstream gene expression or conducting animal experiments using NRF2 knockout rats.

## Conclusion

GXBD could improve cardiac function and reduce pathological damage to myocardial tissue in rats. The mechanism might be related to the PI3K/AKT/NRF2 signaling pathway to improve mitochondrial function. This study suggested that GXBD may be a potential traditional Chinese medicine against MI, and this project provided scientific support for the clinical application of GXBD.

## Data Availability

The original contributions presented in the study are included in the article/Supplementary Material, further inquiries can be directed to the corresponding authors.
