# Supplementary material for: Integrating network pharmacology and experimental validation to investigate the mechanism of Gualou Xiebai Banxia decoction against myocardial ischemia
Source: Front Cardiovasc Med. 2025 Apr 22;12:1512791. doi: 10.3389/fcvm.2025.1512791 (PMC12052758; doi:10.3389/fcvm.2025.1512791)
Supplement: Supplementary file 1 [file Image1.pdf]

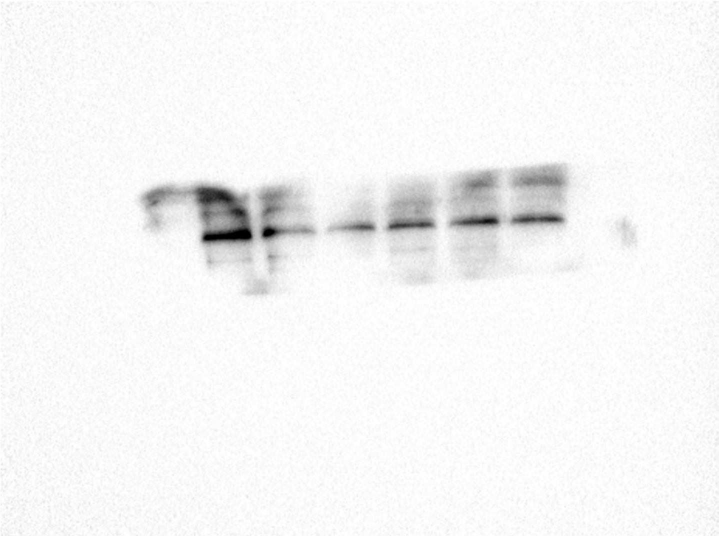

MFN2 86 kDa

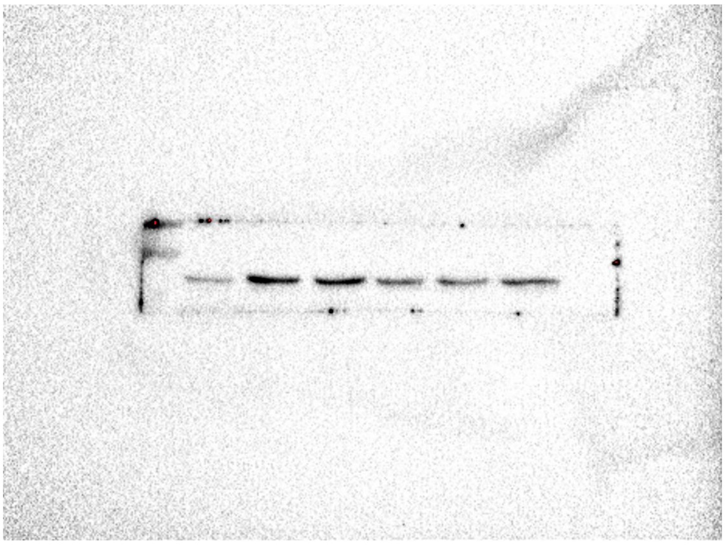

DRP1 82 kDa

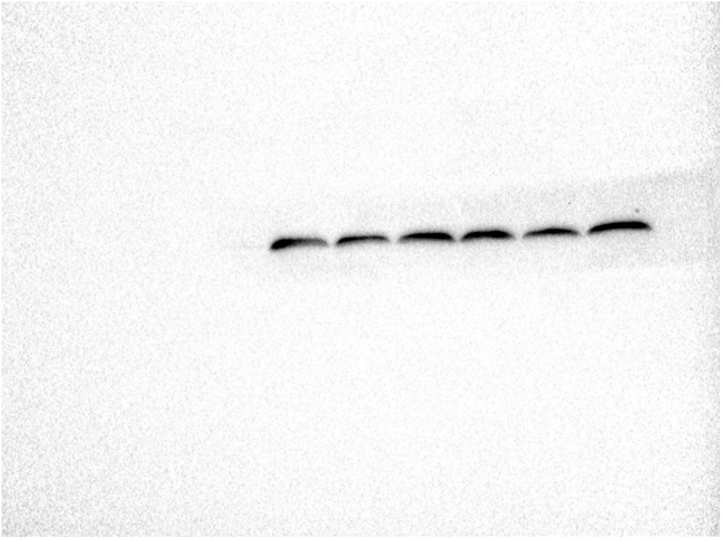

β-actin 42 kDa

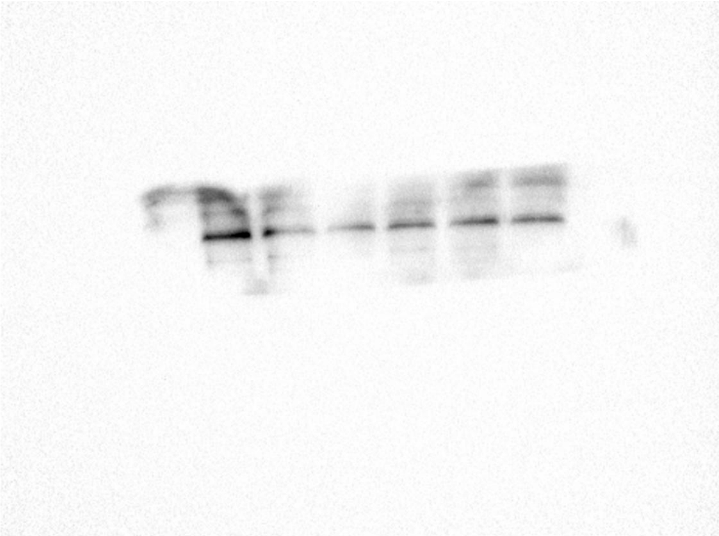

p-PI3K 85 kDa

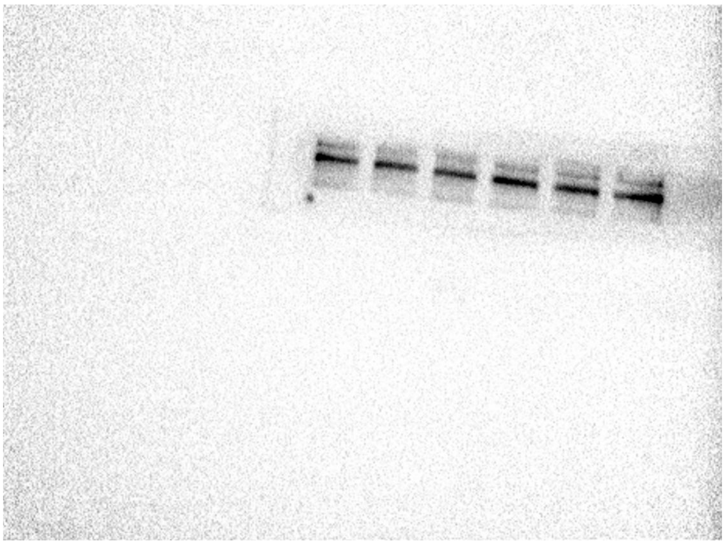

PI3K 85 kDa

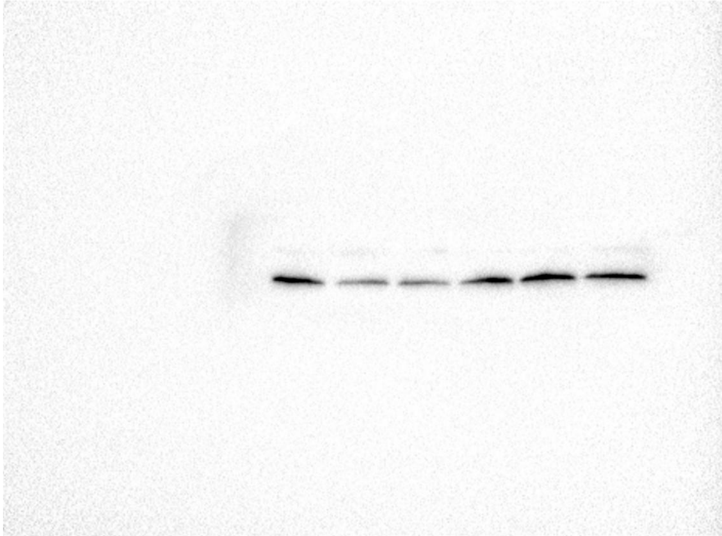

p-AKT 60 kDa

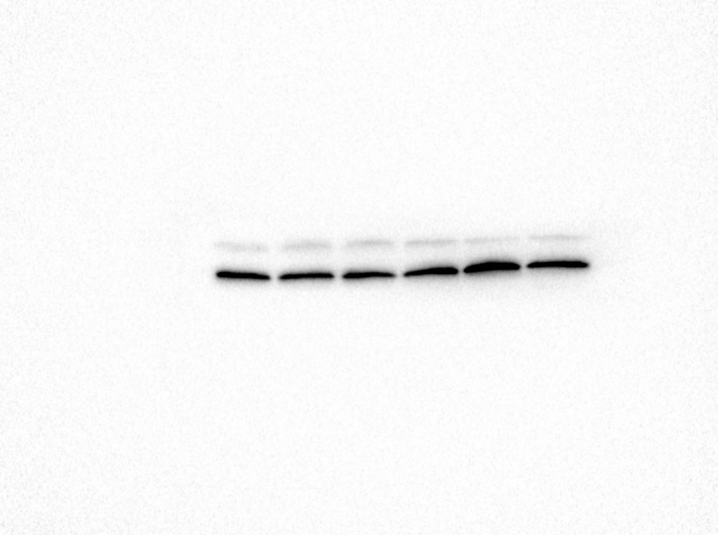

AKT 60 kDa

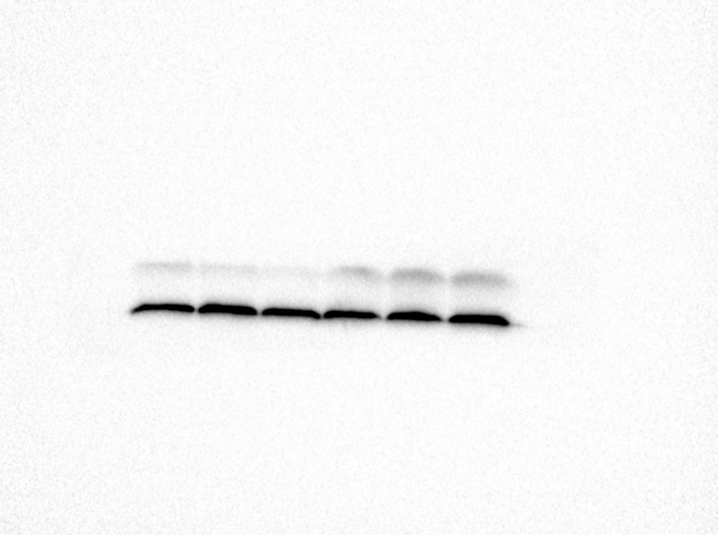

β-actin 42 kDa

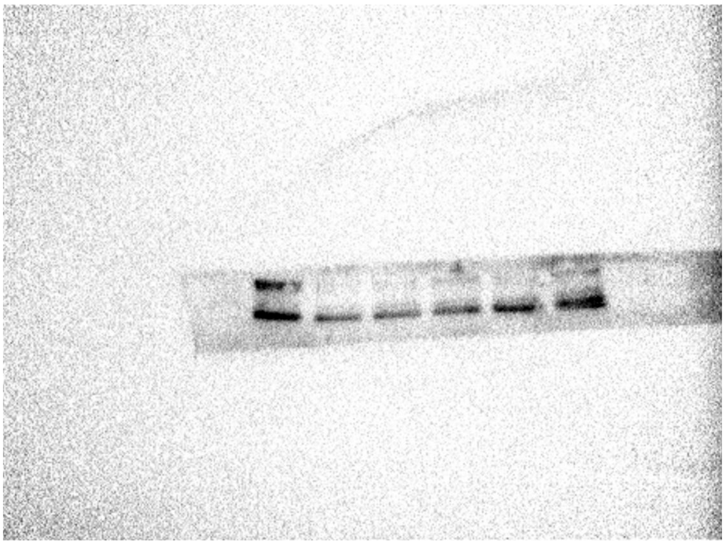

Nuclear NRF2 110 kDa

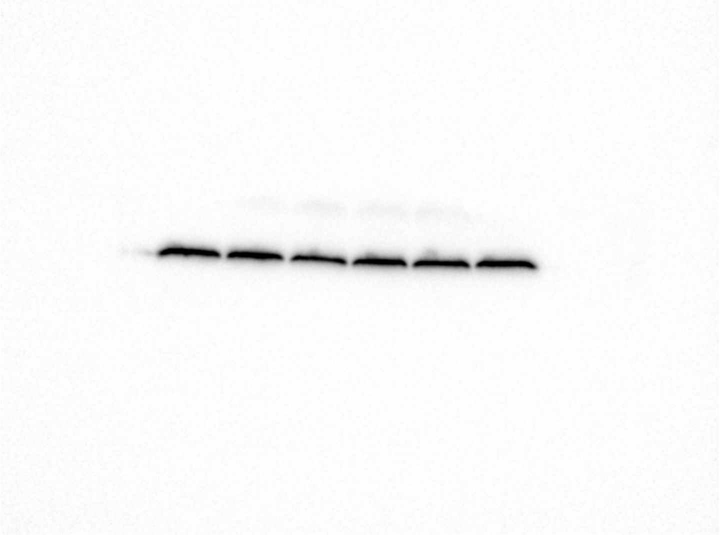

Lamin B 68 kDa

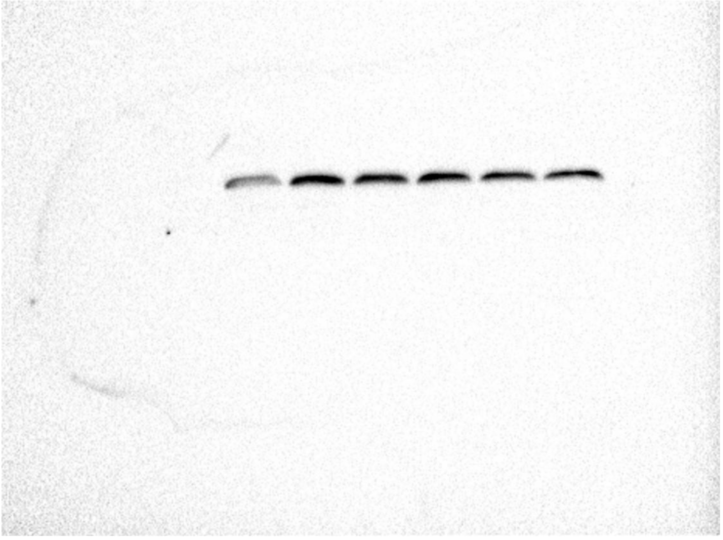

Cytoplasmic NRF2 110 kDa

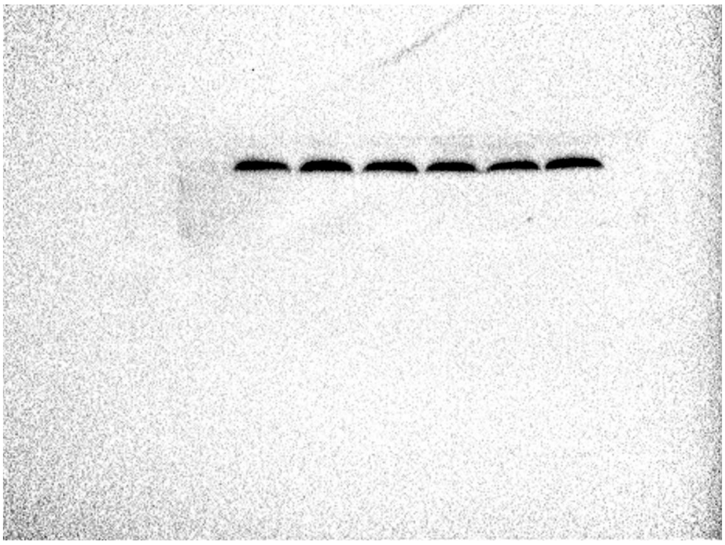

β-actin 42 kDa

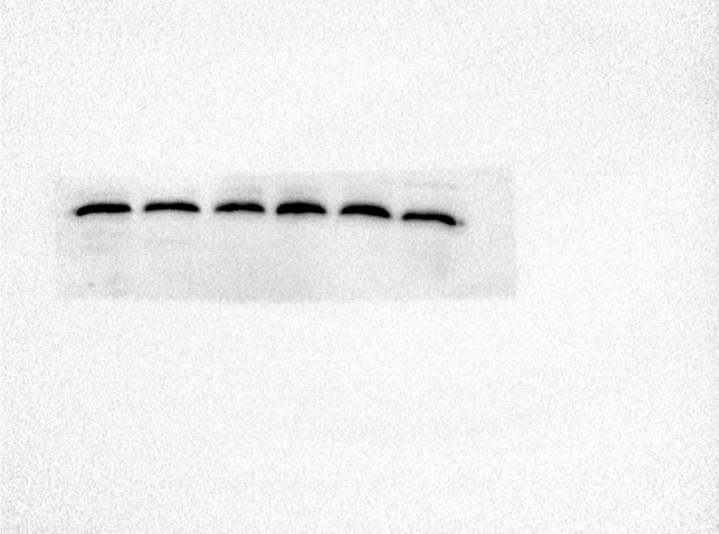

NRF2 110 kDa

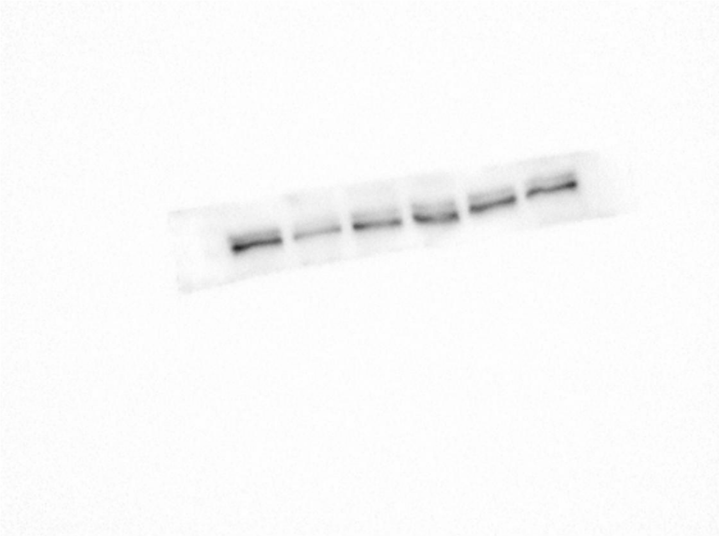

HO-1 32 kDa

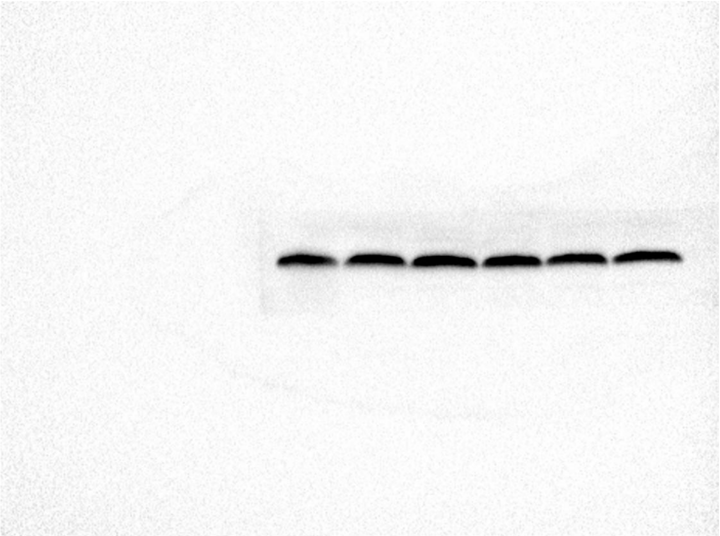

β-actin 42 kDa

Supplementary figure 1. Original western blots from which the corresponding bands were cropped in figure 1.
